# Multicellular dynamics and wealth distribution in bacteria

**DOI:** 10.1038/s44320-024-00056-3

**Published:** 2024-07-15

**Authors:** Kyle R Allison

**Affiliations:** Division of Infectious Diseases, Department of Medicine, Emory University School of Medicine, Atlanta, GA USA

**Keywords:** Evolution & Ecology, Microbiology, Virology & Host Pathogen Interaction

## Abstract

KR Allison discusses a dynamic model of multicellular “patches” of bacteria upon antibiotic treatment to show beneficial community interactions support their collective survival as reported by Şimşek et al in this issue of *Molecular Systems Biology*.

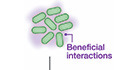

## Teaser

In a variety of infections, bacteria form multicellular communities which contribute to virulence and antibiotic tolerance. The molecular biology of such communities is actively studied, but our understanding of them remains incomplete. In this issue, Şimşek et al have developed and experimentally validated a dynamic model for community formation and revealed general insights into bacterial collective survival during antibiotic exposure.

As documented by *Forbes Magazine*, there are now more billionaires than ever and they are richer than ever too. This is great news for billionaires and perhaps for research institutions, which regularly benefit from the generosity of wealthy individuals. But it also serves as a reminder that the distribution of wealth among humans is not equal. The degree of this *inequality* is often quantified by the Gini coefficient, which measures the difference between an idealized distribution of perfect equality and a real one. As wealth becomes concentrated in fewer hands, i.e. increasing inequality, the Gini coefficient increases, a phenomenon sometimes expressed as the “rich get richer”. It turns out, bacteria do this too. In this issue of *Molecular Systems Biology*, Emrah Şimşek and colleagues from Lingchong You’s Lab developed a versatile dynamical model and applied it—along with the Gini coefficient—to distributions of bacterial communities, revealing that rich multicellular communities get richer in the face of antibiotic treatment (Şimşek et al, 2024). Their study has implications for fighting bacterial infections and provides a framework for exploring multicellular dynamics. Despite such intriguing wealth distributions, however, *Forbes* probably won’t be publishing a “Bacteria List” anytime soon.

Bacteria can grow as individual cells but often create multicellular communities when infecting patients. Multicellular communities tolerate antibiotic treatment, therefore contributing to the spread and recurrence of infections (despite individual cells lacking antibiotic resistance from the clinical standpoint). This in part has motivated study of surface-attached communities known as biofilms. But the connection between biofilms and infections is shifting: properties attributed to large (macroscopic) surface-attached biofilms are being reported in small (microscopic) multicellular aggregates that aren’t attached to a surface. Moreover, such microscopic, un-attached aggregates are increasingly recognized in infections—including by *Escherichia coli*, *Pseudomonas aeruginosa*, and *Mycobacterium tuberculosis* (just to get the list started)—and are changing the conversation on biofilms and infectious diseases (Sauer et al, 2022). Winning the fight against bacterial infections will likely require understanding how these aggregates form and how they contribute to virulence and antibiotic tolerance.

Dynamics, the temporal behavior of systems, characterize living organisms and contextualize properties like robustness and emergence. Cells are pretty complicated at the molecular scale, and dynamical modeling provides a useful basis that doesn’t require knowing all the molecular details. In fact, dynamics can even clarify such details, and demonstrations of this point (Alon et al, 1999) helped jump-start Systems Biology. Attention has turned to the dynamic formation of multicellular “patches,” so-called as bacteria often form spatially-distributed patches in infections, instead of uniformly covering the available space. But “patchy dynamics” (alternatively “multicellular dynamics,” “aggregate dynamics,” etc) don’t only occur in infections, they can also be studied in the lab. As illustrated by an excellent previous study in soil bacterium *Bacillus subtilis* (Ratzke and Gore, 2016), combining dynamical modeling and experimentation to patchy dynamics can produce general insights. However, patchy dynamics during antibiotic treatment, though important, are largely unexplored and it is unclear what statistical measure can best capture distributions in patch size.

Şimşek et al have compellingly addressed these gaps. First, they developed a straightforward differential-equations model that incorporates nutrient and antibiotic diffusion, cell dispersion and motion (or its lack due to cell-cell adhesion), and beneficial community interactions which can serve as a positive feedback loop acting over short-length scales (in this case a self-produced antibiotic-degrading enzyme). This model is simulated and the variation in patch size is quantified using the Gini coefficient. As the Gini coefficient includes all members of a distribution and is agnostic to underlying mechanisms, it is an astute choice and should be considered for related studies in the future. From the simulations, the authors elucidate a number of predictions regarding how patchy dynamics depend on relative values of the parameters, e.g., patchiness emerges at intermediate antibiotic concentrations and it also requires beneficial community interaction, referred to as “collective survival.” Next, the authors experimentally verify many of the model predictions in petri-dish agar experiments. To do so they use an elegant gene circuit previously developed to produce altruistic cell death (Tanouchi et al, 2012). These cells create an antibiotic-degrading enzyme but don’t export it. The enzyme only provides a community benefit when a cell is killed by antibiotic and releases its contents. Their experiments demonstrate the versatility and value of their dynamical approach, and include some clever examples where the effect of motility (bacterial swimming) is determined simply by tuning the concentration of agar.

**Figure 1:**
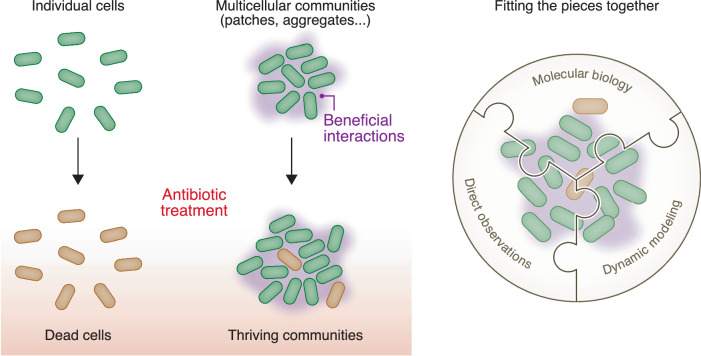
Collective survival of bacterial communities. (Left) Antibiotic exposure kills individual cells which lack antibiotic resistance genes. However, in multicellular communities (including patches, aggregates, biofilms, etc) beneficial interactions can enable the same bacteria to thrive. Different species can employ a variety of molecular mechanisms to produce such collective survival. Using dynamic modeling, Şimşek et al show that local collective survival enables multicellular communities “rich” in beneficial interactions to grow “richer,” thereby accounting for “patchiness,” i.e., the spatial distribution in community size. Their study indicates that the dynamics of multicellular survival may be general, despite the underlying molecular mechanisms being diverse, and provides a straightforward model for contextualizing molecular-scale insights. (Right) Multicellular communities play critical roles in variety of bacterial infections, including driving virulence and antibiotic tolerance. Deciphering these roles will advance our approaches to infectious diseases, and will require dynamic models (like Şimşek et al’s), molecular insights, and direct observations of bacterial behavior. Without any one of these pieces, the picture will remain incomplete. Source data are available online for this figure.

Patchy dynamics are of course motivated by bacterial disease, and the *E. coli* experiments are meant more to test the model than to represent a particular infection. Hence, the authors extended their approach to investigate *P. aeruginosa* patchy dynamics during antibiotic treatment. *P. aeruginosa* is a particularly bad bacterium and causes antibiotic-tolerant wound infections and chronic lung infections in people with Cystic fibrosis (PwCF). Its biofilms and aggregates have been thoroughly studied, though its behavior has been puzzling from the molecular-biology perspective as its genetic variation (even whether or not it has antibiotic resistance) does not fully explain its infectivity and tolerance in PwCF (Vanderwoude et al, 2024). In addition, *P. aeruginosa* CF-isolates can form different types of microscopic aggregates, dependent on the O-antigen, in conditions that include polymers to mimic infections (Azimi et al, 2021). In fact, the Gini coefficient has been applied to characterize microscopic aggregates of *P. aeruginosa* CF-isolates (Cai et al, 2019), again recommending its value. Although the molecular mechanisms underlying collective survival in *P. aeruginosa* are not fully clear, Şimşek et al demonstrate their model is applicable to patchy dynamics here too, and the findings largely mirror their findings in *E. coli*. This is perhaps surprising: *P. aeruginosa*’s mechanism of collective survival is certainly different from *E. coli*’s, and similar dynamics for differing molecular mechanisms then illustrates the general utility of the model Şimşek et al have developed. There may also be more to the story. The patchy dynamics of *B. subtilis*, which has different natural habitats and mechanisms of collective survival, are also remarkably similar (Ratzke and Gore, 2016). It appears, in stressful environments, any multicellular interaction that creates a collective benefit enables community survival and patch formation (Fig. 1, left). If these collective behaviors are easy for bacteria to implement, survival would depend less on their precise genetics and more on their multicellular interactions. Any collective benefit might then drive the transition to multicellularity, and it’s intriguing to consider the problems of *P. aeruginosa* infections from this perspective.

Naturally, models are useful but imperfect and can “fail” when evidence changes scale. In the future, investigating *microscopic* bacterial communities will bring the *continuous* differential equations of Şimşek et al,’s model up against the *quantal* behavior of individual cells. This could create issues. For example, their study indicates bacterial motility reduces patchiness at the macroscopic scale, which is consistent with previous findings (Ratzke and Gore, 2016). However, direct observation of individual *E. coli* cells has shown that motility facilitates aggregate formation at the microscopic scale (Laganenka et al, 2016), behavior later linked to *E. coli*’s colonization of the intestine, its primary habitat (Laganenka et al, 2023). At the *macroscopic* scale, microscopic aggregates are either imperceptible or perhaps obscured by non-aggregated, swimming cells. Rather than a drawback of the model, however, such complications help focus research and can lead to important mechanistic insights.

Applying the authors’ approach—both the dynamic model and Gini coefficient—at the microscopic scale could help clarify the diverse molecular findings accumulating on bacterial community formation. Molecular biology, dynamic modeling, and direct observations are complementary approaches, each of which provides unique insights for completing the picture of “bacterial multicellularity.” Fitting all of these pieces together will be critical for understanding the roles of multicellular communities in infections (Fig. 1, right), including how they regulate genes for virulence and antibiotic tolerance. Recent findings appear to point in this direction, including examples that dynamic multicellular aggregation can lead to previously-unrecognized bacterial life cycles (Schwartzman et al, 2022).

### Supplementary information

Source data Fig. 1
